# Evaluating the therapeutic potential of moxibustion on polycystic ovary syndrome: a rat model study on gut microbiota and metabolite interaction

**DOI:** 10.3389/fcimb.2024.1328741

**Published:** 2024-04-11

**Authors:** Yong Lin, Huiling Zeng, Jieying Lin, Yiwei Peng, Xueyun Que, Lijun Wang, Ling Chen, Ni Bai

**Affiliations:** ^1^ Department of Traditional Chinese Medicine Specialty Diagnosis and Treatment, Xiamen Hospital of Traditional Chinese Medicine, Xiamen, China; ^2^ Department of Traditional Chinese Medicine, School of Medicine, Xiamen University, Xiamen, China; ^3^ College of Acupuncture and Moxibustion, Fujian University of Traditional Chinese Medicine, Fuzhou, China; ^4^ Dongzhimen Hospital, Beijing University of Chinese Medicine, Beijing, China

**Keywords:** moxibustion, polycystic ovary syndrome, 16S rDNA sequencing, ^1^H NMR, DHEA, microbiota, metabolites

## Abstract

Polycystic ovary syndrome (PCOS) is a common systemic disorder related to endocrine disorders, affecting the fertility of women of childbearing age. It is associated with glucose and lipid metabolism disorders, altered gut microbiota, and insulin resistance. Modern treatments like pioglitazone, metformin, and spironolactone target specific symptoms of PCOS, while in Chinese medicine, moxibustion is a common treatment. This study explores moxibustion’s impact on PCOS by establishing a dehydroepiandrosterone (DHEA)-induced PCOS rat model. Thirty-six specific pathogen-free female Sprague-Dawley rats were divided into four groups: a normal control group (CTRL), a PCOS model group (PCOS), a moxibustion treatment group (MBT), and a metformin treatment group (MET). The MBT rats received moxibustion, and the MET rats underwent metformin gavage for two weeks. We evaluated ovarian tissue changes, serum testosterone, fasting blood glucose (FBG), and fasting insulin levels. Additionally, we calculated the insulin sensitivity index (ISI) and the homeostasis model assessment of insulin resistance index (HOMA-IR). We used 16S rDNA sequencing for assessing the gut microbiota, ^1^H NMR spectroscopy for evaluating metabolic changes, and Spearman correlation analysis for investigating the associations between metabolites and gut microbiota composition. The results indicate that moxibustion therapy significantly ameliorated ovarian dysfunction and insulin resistance in DHEA-induced PCOS rats. We observed marked differences in the composition of gut microbiota and the spectrum of fecal metabolic products between CTRL and PCOS rats. Intriguingly, following moxibustion intervention, these differences were largely diminished, demonstrating the regulatory effect of moxibustion on gut microbiota. Specifically, moxibustion altered the gut microbiota by increasing the abundance of *UCG-005* and *Turicibacter*, as well as decreasing the abundance of *Desulfovibrio*. Concurrently, we also noted that moxibustion promoted an increase in levels of short-chain fatty acids (including acetate, propionate, and butyrate) associated with the gut microbiota of PCOS rats, further emphasizing its positive impact on gut microbes. Additionally, moxibustion also exhibited effects in lowering FBG, testosterone, and fasting insulin levels, which are key biochemical indicators associated with PCOS and insulin resistance. Therefore, these findings suggest that moxibustion could alleviate DHEA-induced PCOS by regulating metabolic levels, restoring balance in gut microbiota, and modulating interactions between gut microbiota and host metabolites.

## 1 Introduction

Polycystic ovary syndrome (PCOS) stands as the most prevalent endocrine and metabolic disorder among women of childbearing age. Its clinical manifestations encompass hyperandrogenism, ovulatory dysfunction, and the characteristic polycystic ovary morphology (PCOM). The prevalence of PCOS is about 26%, which comes with appearance changes like hairy, obese and black acanthosis (Bayona et al., 2022). Beyond these external manifestations, alterations in oocyte and endometrial quality directly impact the reproductive outcomes and fertility potential (Palomba et al., 2017) of women with PCOS, significantly elevating the risk of infertility (Palomba, 2021; Palomba et al., 2021). Moreover, even upon successful conception, individuals with PCOS are at an increased risk for pregnancy-related complications, including gestational diabetes, pregnancy-induced hypertension, and preterm birth (Palomba et al., 2015). Consequently, PCOS contributes to substantial physical and mental discomfort and fertility anxiety to women, is an urgent medical problem to be solved in the field of female reproductive endocrinology (Chen et al., 2018; Hamilton et al., 2019).

PCOS is widely regarded to be closely related with glucose and lipid metabolism disorders, often combined with insulin resistance (IR), hyperinsulinemia, obesity and other diseases (Liao et al., 2021). Among them, insulin resistance is one of the pivot characteristics of PCOS. Studies have shown that 60% -80% of PCOS patients are accompanied by varying degrees of insulin resistance (Wang et al., 2019; Hu et al., 2020; Amisi, 2022). Therefore, in the clinical treatment of PCOS, it is of vital necessity to take the regulation of glucose and lipid metabolism into account. As we all know, metformin is a powerful weapon to regulate the disorder of glucose and lipid metabolism in patient with PCOS, which acts an assistant role in the treatment of PCOS (Palomba et al., 2009; Liu et al., 2023b). However, prolonged metformin use leads to produces gastrointestinal side effects such as diarrhea, abdominal pain, abdominal distension and taste disorder (Ramu et al., 2022). In contrast, moxibustion therapy, an usual treatment of PCOS in traditional Chinese medicine, achieves therapeutic effects through warm stimulation and drug stimulation of body surface acupoints. It is characterized by its non-invasive nature, minimal adverse effects, simplicity of operation, and effectiveness (Xu et al., 2021).

Currently, the exact pathogenesis of PCOS remains incompletely understood. However, numerous studies have shown that the disorder of intestinal flora is closely related to PCOS, and can even directly affect the incidence of PCOS (Guo et al., 2022; Liu et al., 2023a). Intestinal tract is known as the ‘ second brain ‘ of human beings. The metabolism of intestinal flora has important feedback-and-regulation effects on the physiological functions of the body (Reutov and Sorokina, 2022). Research has demonstrated that metformin can ameliorate the metabolic and endocrine profiles of PCOS patients, as well as the diversity and abundance of intestinal flora (Gan et al., 2023). Therefore, in this experiment, we employed HE staining to assess the impact of moxibustion on the ovarian morphology of PCOS rats, and then the effect of moxibustion on PCOS was observed from the aspects of body weight, serum testosterone level, fasting blood glucose (FBG), serum insulin level, insulin sensitivity index (ISI) and insulin resistance level (HOMA-IR). Combined with metabolomics based on nuclear magnetic resonance (NMR) technology and microbiome based on 16S rRNA technology, the effects of moxibustion on PCOS metabolomics and microbiome were studied to explore the potential mechanism of moxibustion in the treatment of PCOS, and to provide some experimental basis for moxibustion in the treatment of PCOS.

## 2 Materials and methods

### 2.1 Animals

36 Female Sprague-Dawley (SD) rats aged 22 to 23 days were purchased from Beijing Vital River Laboratory Animal Center (Beijing, China). The ethical approval for this study was obtained from the Xiamen University Experimental Animal Center Ethics Committee (permit number XMULAC20230172). The rats were housed in a Specific Pathogen-Free (SPF) environment at the Xiamen University Laboratory Animal Center, maintaining a room temperature ranging from 22°C to 26°C and humidity levels at 60%-70%. The rats were subjected to a 12-hour light/dark. Random allocation was performed to divide the 36 rats into four groups, with 9 rats in each group: CTRL, PCOS, MBT, and MET.

### 2.2 DHEA-induced PCOS rat model

Prior to commencing the experiments, the rats underwent a one-week acclimation phase during which they had unrestricted access to water. PCOS modeling was induced in all rats by daily subcutaneous injections of 60 mg/kg of DHEA (dissolved in 0.2 ml of sesame oil) (Roy et al., 1962; Paixão et al., 2017), except for the CTRL group. The rats in the CTRL group received daily injections of 0.2 ml of sesame oil.

### 2.3 Treatment

**Figure 1:**
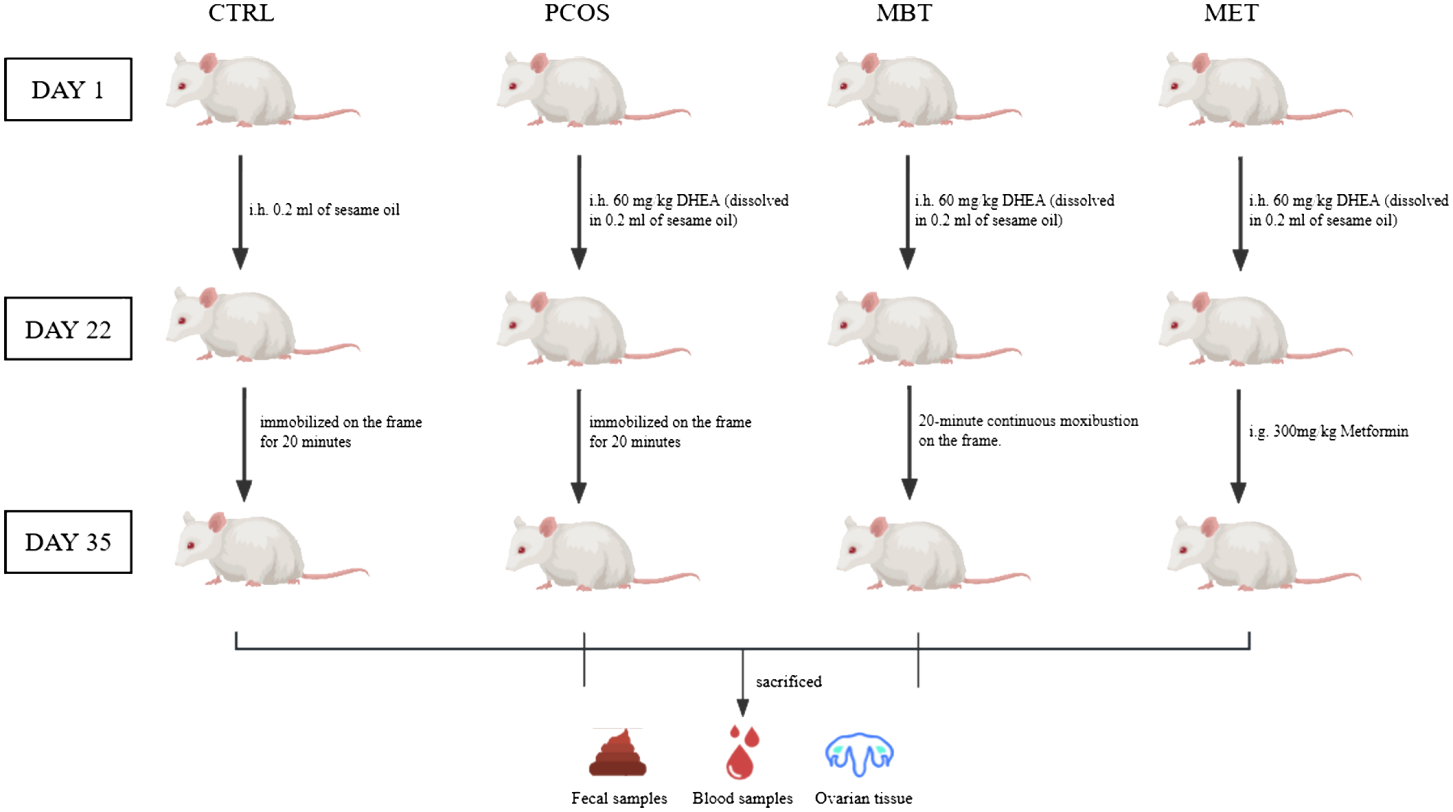
Experimental grouping and procedure.

After the PCOS modelling, the rats in the CTRL and PCOS groups were only immobilized on the frame for 20 minutes daily over a 14-day period, without receiving any additional treatment. In the MBT group, the rats were immobilized on the frame and received daily moxibustion treatment at Guanyuan acupoint (CV4) for 20 minutes over the same 14-day period. The moxibustion was carried out using special animal-specific moxa sticks (dimensions: height 5 mm, diameter 5 mm, “Han Medicine,” Nanyang, China), held 2 cm above the CV4. The selection of CV4 in the Ren Meridian for moxibustion treatment was based on the guidelines outlined in “Chinese Veterinary Acupuncture and Moxibustion” (Liu, 2013). As for the MET group, the rats were administered metformin through daily gavage at a dose of 300mg/kg for 14 days (**Figure 1**).

### 2.4 Sample collection

Throughout both the modelling and treatment phases, the rats’ body weight was diligently monitored on a daily basis. Following completion of the treatment, the rats underwent a 12-hour fasting period, during which a final body weight measurement was conducted before euthanizing all the rats for further analysis. The ovaries were collected for histopathological examination, while blood samples were obtained for subsequent biochemical analysis. Moreover, fecal samples were collected and transferred into cryogenic storage tubes, frozen in liquid nitrogen, and preserved at -80°C for subsequent ^1^H NMR-based metabolomics testing and 16S rDNA analysis.

### 2.5 H&E staining of ovarian tissue

Ovarian tissue samples were collected and washed with sterile 0.9% NaCl solution on aseptic equipment. Subsequently, the tissues were immersed in a 4% paraformaldehyde solution for 48 hours. Sections of paraffin, each with a thickness of 5µm, were meticulously prepared, subjected to dewaxing, and then stained using the haematoxylin and eosin (H&E) method. These stained sections were observed under a Leica Aperio Versa 200 microscope in Tokyo, Japan, to assess the extent of pathological damage.

### 2.6 Measurement of serum biochemical markers

Blood samples were collected from rats and coagulated for 40 minutes. After centrifugation at 3,000 r for 20 minutes, the serum was stored at −80°C. Serum levels of FBG, fasting insulin, and testosterone were measured using an enzyme-linked immunosorbent assay (ELISA) following the manufacturer’s protocol. The testosterone ELISA kit (E-EL-0155c) and insulin ELISA kit (E-EL-M2614c) were procured from Elabscience, while the blood glucose kit (JL-T1253) was obtained from Jianglai Biotechnology. The coefficient of variation (CV) for all utilized assay kits was less than 10%. For detailed parameters, refer to **Tables 1**, **2**.

**Table 1:** CV for Testosterone Assay Kit.

	Intra-assay Precision			Inter-assay Precision		
Sample	1	2	3	1	2	3
n	20	20	20	20	20	20
Mean(ng/mL)	1.33	5.22	10.88	1.54	5.73	10.02
Standard deviation	0.11	0.35	0.46	0.14	0.35	0.68
CV(%)	8.13	6.72	4.27	9.23	6.18	6.75

**Table 2:** CV for Insulin Assay Kit.

	Intra-assay Precision			Inter-assay Precision		
Sample	1	2	3	1	2	3
n	20	20	20	20	20	20
Mean(ng/mL)	1	2.3	8.5	1	2.3	8.7
Standard deviation	0.1	0.1	0.4	0.1	0.1	0.3
CV(%)	10	4.35	4.71	10	4.35	3.45

### 2.7 16S rDNA gene sequencing

Fresh fecal samples collected from the sacrificial rats were aseptically placed in sterile EP tubes and stored at -80°C for subsequent processing. Total bacterial DNA was extracted from the fecal samples using the FastDNA^®^SPIN Kit for Soil (Omega Bio-tek, Norcross, GA, USA) following the manufacturer’s instructions. The concentration and purity of the DNA were assessed using a NanoDrop 2000 UV-vis spectrophotometer (Thermo Scientific, Wilmington, USA). Subsequently, the hypervariable region V3-V4 (Liu et al., 2016) of the bacterial 16S rDNA gene was amplified using an ABI GeneAmp^®^ 9700 PCR thermocycler (ABI, CA, USA). The selected primers were 338F (ACTCCTACGGGAGGCAGCAG) and 806R (GGACTACHVGGGTWTCTAAT). The PCR amplification of the 16S rRNA gene involved an initial denaturation at 95°C for 3 minutes, followed by 30 cycles of denaturation at 95°C for 30 seconds, annealing at 55°C for 30 seconds, extension at 72°C for 45 seconds, and a final extension at 72°C for 10 minutes. The PCR reactions were conducted in triplicate, and the resulting PCR products were combined and purified using the AxyPrep DNA Gel Extraction Kit (Axygen Biosciences, Union City, CA, USA) in accordance with the manufacturer’s instructions. DNA quantification was carried out using a Quantus™ Fluorometer (Promega, USA), and the purified pooled samples underwent sequencing analysis on the Illumina MiSeq platform (Illumina, USA).

### 2.8 Samples preparation and ^1^H NMR experiments

Metabolites in faeces were analyzed using ^1^H NMR-based metabonomics. Fecal samples weighing 50 to 60 mg were kept on ice and subsequently homogenized in 1 ml of PBS (0.1 M) containing 50% D_2_O. The homogenization process involved vortexing for 1 minute. Afterwards, the samples underwent two freeze-thaw cycles using liquid nitrogen and were then centrifuged at 4°C and 12,000 r for 10 minutes. The resulting supernatants were then transferred to new microcentrifuge tubes (2 ml). The pellets were reconstituted with 0.6 ml of PBS solution, vortexed for 30 seconds, and then centrifuged once again at 4°C and 12,000 r for 10 minutes. The supernatants were combined, and 40 μl of D_2_O containing disodium terephthalate (Wang et al., 2022) was added. After further centrifugation at 4°C and 16,000 r for 10 minutes, the resulting supernatants (0.55 ml) were transferred to 5 mm NMR tubes.

The NMR analysis was performed using a 500 MHz Bruker spectrometer (Bruker AV500, Bruker Corporation, Switzerland) employing the Carr Purcell Meiboom-Gill (CPMG) pulse sequence. The specific scanning parameters were configured as follows: a spectral width of 12.019 kHz, a relaxation time of 320 ms, 32 scanning times, FID conversion, a line broadening factor (LB) of 0.3 Hz, a pulse width (PW) of 30°C (12.7 μs), and a relaxation delay (RD) of 1.0 s. Following the acquisition of ^1^H NMR spectra with the Bruker NMR spectrometer, metabolite identification was conducted using our team’s NMR metabolites database, published literature, and chemical shift databases such as BMRB (http://www.bmrb.wisc.edu/Metabolomics/) and HMDB (http://www.hmdb.ca/).

### 2.9 Data processing

The processing of fecal samples, encompassing signal denoising, phase correction, and baseline adjustments, was performed using MestReNova version 9.0.1, developed by Mestrelab Research in Santiago de Compostela, Spain. The spectra were standardized by aligning them to their peak values, setting the reference peak of the internal standard at 7.88 ppm. Subsequently, the spectra were segmented into intervals of 0.01 ppm within the range of δ 0.6–9.5 ppm, with the exclusion of the water peak falling within the range of δ 4.70–4.90 ppm. To account for differences in sample concentrations, integral values from each spectrum were normalized relative to the sum of all integrals within that spectrum, facilitating subsequent multivariate analysis. The spectral data were imported into SIMCA-P version 14.1 software, developed by Umetrics in Sweden, and Pareto-scaling (Par) was applied to reduce noise and eliminate artifacts within the model. Intergroup separation was evaluated using Partial Least Squares Discriminant Analysis (PLS-DA). Subsequently, potential variables were analyzed through Orthogonal Partial Least Squares Discriminant Analysis (OPLS-DA), following an assessment of the OPLS-DA model’s quality based on model goodness of fit (R2) and prediction ability (Q2). Endogenous differential metabolites were identified based on their importance in the project (VIP > 1), log2 fold change (|log2FC| > 0.5), and the independent sample t-test (*p* < 0.05).

### 2.10 Statistical analysis

All statistical analyses were performed using GraphPad Prism 9.0 software (GraphPad Software Inc, San Diego, CA, USA). One-way analysis of variance (ANOVA) was employed to assess variances in each variable among the four groups, and the data were visually presented in plots. The values are reported as the mean ± standard error. Statistical significance was established at a significance level of *p* < 0.05. To investigate the relationship between fecal metabolite levels and the relative abundance of genera, Spearman correlation analysis was conducted using the correlation test function from the R package “stats.” Correlation analysis was limited to those genera (*p <* 0.05) and metabolites (*p* < 0.05, VIP > 1, |log_2_FC| > 0.5) that displayed statistically significant distinctions among the groups.

## 3 Results

### 3.1 Moxibustion improves ovarian dysfunction in PCOS rats

**Figure 2:**
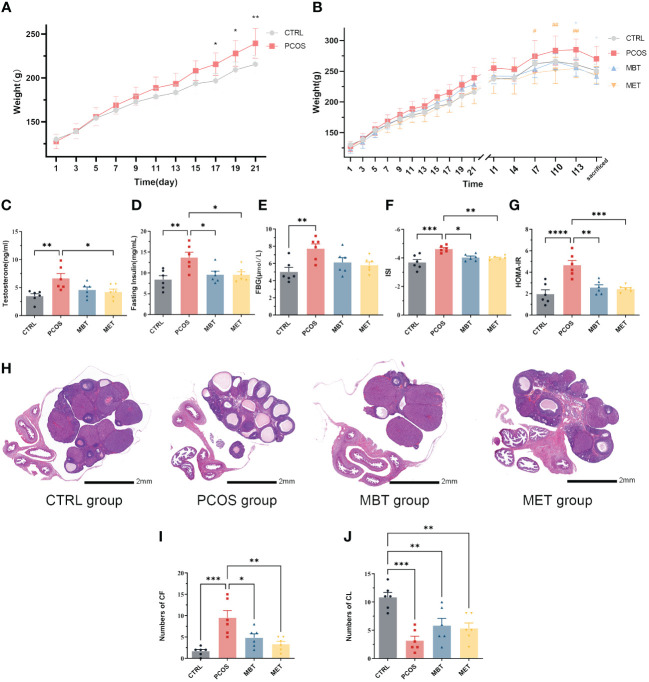
Moxibustion alleviates ovarian dysfunction and insulin sensitivity in DHEA-induced PCOS rats. **(A)** Body weight changes in DHEA-Induced Rats. **(B)** Changes in body weight of rats throughout the entire experimental process in each group. * indicates significance (* *p* < 0.05, ** *p* < 0.01) compared with the MBT group, # indicates significance (# *p* < 0.05, ## *p* < 0.01) compared with the MET group. **(C)** Expression of testosterone. **(D)** Expression of fasting insulin. **(E)** Expression of FBG. **(F)** The insulin sensitivity (ISI) index was calculated as follows: 1/[(fasting insulin × fasting glucose) ^ 0.5]. **(G)** The homeostasis model assessment of insulin resistance (HOMA-IR) index was calculated as follows: (fasting glucose) × (fasting insulin]/22.5). **(H)** H&E staining of ovarian tissue. **(I)** The number of cystic follicles (CF). **(J)** The number of corpora lutea (CL). Data are mean ± s.d. n = 6. * *p* < 0.05, ** *p* < 0.01, *** *p* < 0.001, **** *p* < 0.0001.

At the end of the modelling process, notable dissimilarities in body weight were observed between the CTRL and PCOS groups, with a substantial increase in body weight recorded in the PCOS group (**Figure 2A**). As the experimental period concluded, both the MET and MBT groups revealed lowering in body weight compared to the PCOS group (**Figure 2B**).

Serum testosterone levels were assessed. In contrast to the CTRL group, the PCOS group displayed an elevation in testosterone levels. Metformin treatment obviously reduced testosterone levels in PCOS rats, while moxibustion treatment showed no significant impact on serum testosterone levels (**Figure 2C**). Considering the close association between PCOS and metabolic disorders, we evaluated insulin sensitivity within the various groups. Fasting insulin levels displayed a distinct increase in PCOS rats, and both moxibustion and metformin treatments effectively decrease fasting insulin levels in PCOS rats (**Figure 2D**). Although moxibustion and metformin treatments did not show significant differences in FBG levels (**Figure 2E**), rats in the PCOS group demonstrated lower ISI and higher HOMA-IR compared to those in the CTRL group. Both moxibustion and metformin treatments substantially enhanced ISI (**Figure 2F**) and diminished HOMA-IR (**Figure 2G**) in PCOS rats.

Subsequently, we conducted H&E staining to assess ovarian pathological changes in different groups (**Figure 2H**). In the CTRL group, the ovaries displayed follicles at various developmental stages and some fresh corpora lutea. In contrast, the PCOS group exhibited a higher prevalence of cystic follicles and decreasing of corpora lutea compared to the control group. Remarkably, both moxibustion and metformin treatments reduced the number of cystic follicles (**Figure 2I**) and tends to promote corpora lutea formation (**Figure 2J**).

### 3.2 Effect of moxibustion on gut microbiota in PCOS rats

**Figure 3:**
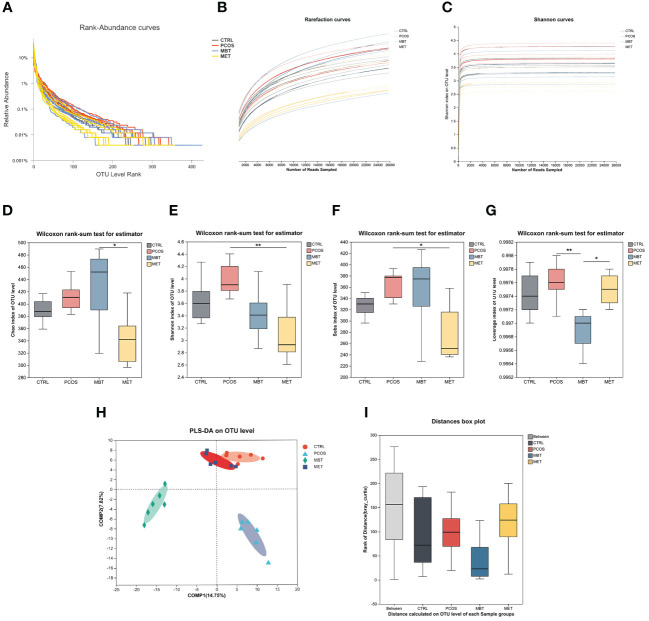
Effect of Moxibustion on diversity of gut microbiota. **(A)** The Rank-Abundance curve of gut microbiota. **(B)** The Rarefaction curve of gut microbiota. **(C)** The Shannon index of gut microbiota. **(D–G)** Alpha diversity of gut microbiota in the rats receiving different treatments, including **(D)** Chao index, **(E)** Shannon index, **(F)** Sobs index, and **(G)** Coverage index. **(H)** PLS-DA analysis of microbiota. **(I)** Anosim analysis was used to detect differences between the groups. Data are mean ± s.d. n = 6. * p < 0.05, ** p < 0.01.

We used 16S rDNA sequencing of fecal samples to investigate the impact of moxibustion on the composition and levels of intestinal microbiota in PCOS rats. To ensure the suitability of fecal samples for sequencing and subsequent analysis, we analyzed rank-abundance curves (**Figure 3A**). Afterward, we assessed bacterial community abundance and diversity using rarefaction (**Figure 3B**) and Shannon curves (**Figure 3C**), as well as four α-diversity indices including Chao (**Figure 3D**), Shannon (**Figure 3E**), Sobs (**Figure 3F**) and coverage index (**Figure 3G**). The sequencing data was seen to be reliable, as there were levelling-off of rarefaction curves once the sequence count reached 10,000. Similar results were observed in the Shannon curves, indicating comprehensive coverage of sample diversity through sequencing. Post-DHEA intervention revealed no significant alterations in α-diversity indices, indicating DHEA’s minimal impact on gut microbiota α-diversity, in contrast to the marked effects of moxibustion and metformin treatments. Noteworthy, the MET group displayed lower Shannon and Sobs indices than the PCOS group (**Figures 3E, F**), and the MBT group’s Coverage index was reduced compared to the PCOS group (**Figure 3G**).

Subsequently, we employed PLS-DA to evaluate the β-diversity of gut microbiota among the different groups (**Figure 3H**). Our findings unveiled substantial distinctions in the overall gut microbiota composition among the CTRL, PCOS, MET, and MBT groups. This suggests distinct gut microbiota profiles among these four groups of rats, particularly between the CTRL and PCOS groups. Additionally, the MET group is more akin to the CTRL group, while the MBT group exhibits clear differences from the other three groups. Anosim analysis also supported the statistical significance of these group differences. Compared with the CTRL group, the PCOS group demonstrated augmented diversity, which suggests an alteration in the gut microbiota due to PCOS. Intriguingly, MBT and MET treatments were associated with lower median diversity values, potentially indicative of their role in moderating the gut microbiota towards the CTRL group’s baseline. The interquartile range overlap between the MBT and MET groups intimates a potential concordance in their effects on gut microbiota diversity (**Figure 3I**).

**Figure 4:**
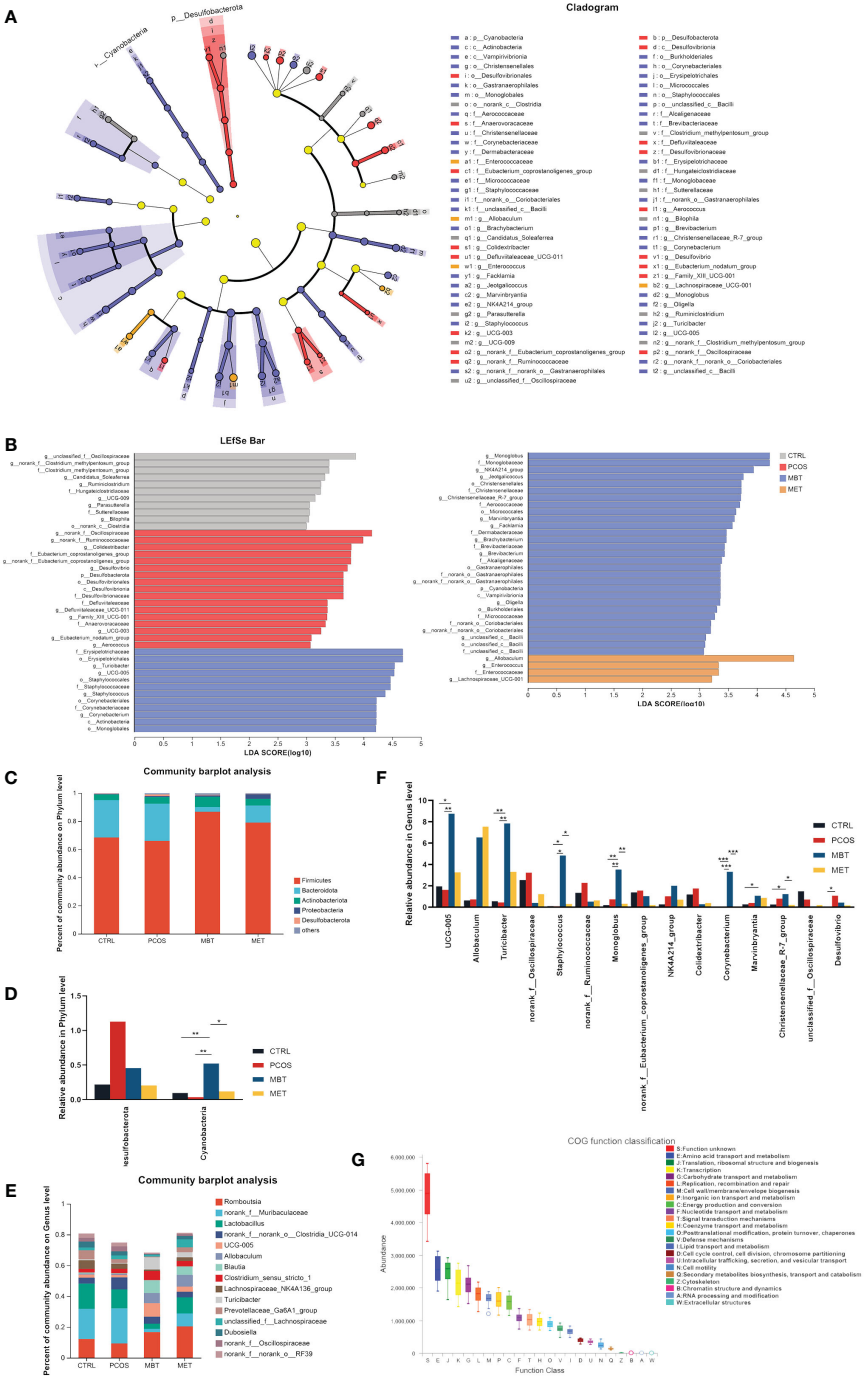
Effect of moxibustion on the taxonomic composition of the gut microbiota. **(A)** Cladograms representing the linear discriminant analysis effect size (LEfSe) results. **(B)** Linear discriminant analysis (LDA) results between different experimental groups. LDA > 3.00 is shown. **(C)** Microbial distribution at the phylum level. **(D)** Significant changes in abundance at the phylum level. **(E)** Microbial distribution at genus level **(F)** Significant changes in abundance of the top 15-genus level. **(G)** Box plot for statistical classification of Clusters of Orthologous Groups (COG) functions. Data are mean ± s.d. n = 6. * *p* < 0.05, ** *p* < 0.01, *** *p* < 0.001.

To further investigate specific taxonomic groups influenced by moxibustion and metformin treatments, we utilized the Linear Discriminant Analysis Effect Size (LEfSe) method (Segata et al., 2011) to analyze validated sequences (**Figure 4A**), and presented the results based on LDA scores greater than 3 (**Figure 4B**). *Clostridia* and *the Clostridium_methylpentosum_group* were enriched in the CTRL group, whereas *Desulfobacterota*, *Anaerovoracaceae*, *Defluviitaleaceae*, and *Alloprevotella* were enriched in the PCOS groups. Additionally, *Cyanobacteria*, *Actinobacteria*, *Bacilli*, *Staphylococcales*, *Christensenellales*, and *Monoglobale* showed enrichment in the MBT group, while the MET group exhibited enrichment of *Enterococcaceae* and *Lachnospiraceae_UCG-001*. *Burkholderiales* were enriched in both the MBT and CTRL groups. These findings suggest that moxibustion may have a more profound impact on reshaping the gut microbiota structure in PCOS rats compared to metformin treatment.

Furthermore, we conducted a comparative analysis of the overall gut microbiota composition among the four groups at the phylum and genus levels. At the phylum level, *Firmicutes*, *Bacteroidota*, and *Actinobacteriota* were identified as the dominant phyla (**Figure 4C**). DHEA intervention led to an increase in *Desulfobacterota* abundance and a decrease in *Cyanobacteria* abundance, while moxibustion therapy seemed to restore the balance in the gut ecosystem by mitigating these changes (**Figure 4D**). At the genus level, *Romboutsia*, *norank_f:Muribaculaceae, Lactobacillus*, *norank_f:norank_o:Clostridia_UCG-014*, and *UCG-005* were the dominant genera (**Figure 4E**). Specifically, DHEA intervention increased the abundance of *norank_f:Oscillospiraceae*, *norank_f:Ruminococcaceae*, *norank_f:Eubacterium_coprostanoligenes_group*, *Colidextribacter*, *Ideonella*, and *Desulfovibrio*, while reducing the abundance of *UCG-005*, *Turicibacter*, and *Staphylococcus*. Notably, moxibustion treatment effectively mitigated these changes (**Figure 4F**).

### 3.3 Regulation of moxibustion on differential fecal metabolites in PCOS rats

**Figure 5:**
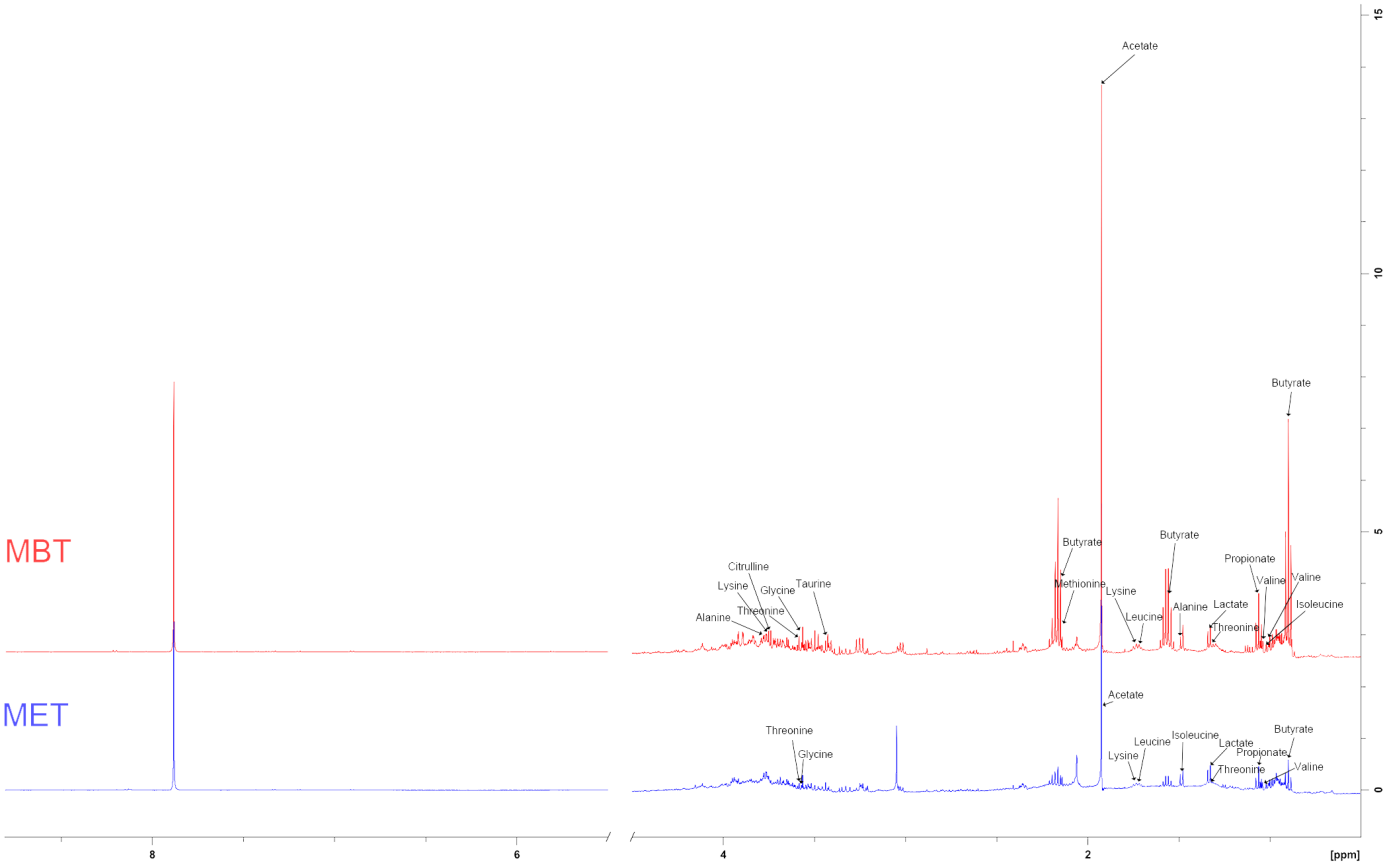
Typical ^1^H NMR spectra of extractive from faeces.

**Figure 6:**
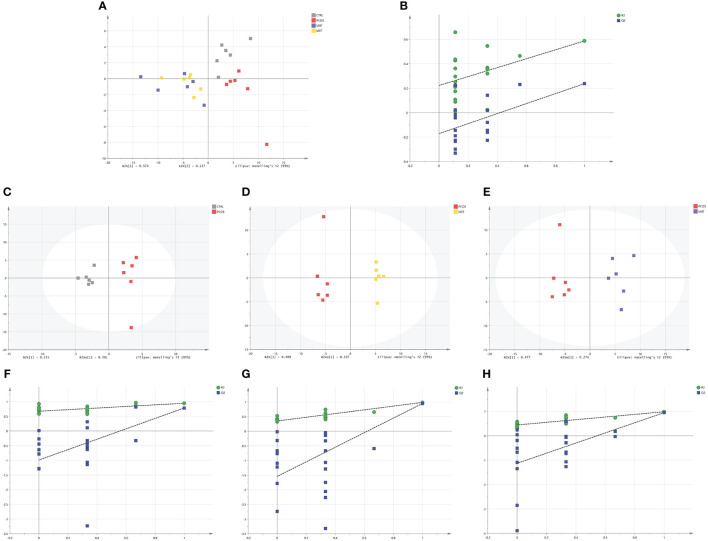
Effect of moxibustion on the metabolite composition of PCOS rats. **(A, B)** PLS-DA analysis of fecal metabolites and corresponding permutation testing (R2X = 0.79 cum, R2Y = 0.574 cum, Q2 = 0.351 cum). **(C, F)** OPLS-DA scores plots and corresponding permutation testing in CTRL and PCOS (R2X = 0.935 cum, R2Y = 0.947 cum, Q2 = 0.785 cum). **(D, G)** OPLS-DA scores plots and corresponding permutation testing in PCOS and MET (R2X = 0.906 cum, R2Y = 0.943 cum, Q2 = 0.896 cum). **(E, H)** OPLS-DA scores plots and corresponding permutation testing in PCOS and MBT (R2X = 0.948 cum, R2Y = 0.985 cum, Q2 = 0.949 cum). Data are mean ± s.d. n = 6.

We conducted a comprehensive investigation of rat fecal metabolic profiles using ^1^H NMR technology. The identification of endogenous metabolites in the spectra was based on existing literature (Lin, 2022), and their authenticity was further confirmed through 2D NMR spectroscopy (**Figure 5**). Metabolic profile model assessment was conducted using PLS-DA, and relationship models among different groups were established under supervised discriminant analysis employing OPLS-DA. The results obtained from the metabolic profiles displayed (**Figures 6A B**) clear inter-group separations for all four groups, indicating distinct metabolic differences among them. Interestingly, the metformin and moxibustion groups showed trends that were closer to the control group, suggesting that moxibustion or metformin treatment might directly or indirectly contribute to the restoration of metabolite levels to normal. Additionally, pairwise comparisons between each group revealed distinct inter-group separations with statistically significant differences, further confirming the reliability of our model’s quality evaluation (**Figures 6C–H**).

Based on the VIP > 1, *p* < 0.05, and |log_2_FC| > 0.5, a total of 14 and 10 endogenous metabolites in fecal tissues were found to be significantly different between the MBT group and the PCOS group, and the MET group and the PCOS group, respectively. These metabolites included Butyrate, Isoleucine, Valine, Propionate, Lactate, Lysine, Leucine, Acetate, Methionine, Taurine, Glycine, Threonine, Citrulline, and Alanine (**Tables 3**, **4**). Notably, these differentially produced endogenous metabolites in the two treatment methods exhibited an upward trend compared to the PCOS group.

**Table 3:** The metabolites identified in feces following moxibustion treatment and their fold change values compared to the PCOS group.

NO.	Formula	Compounds	Abbreviate	δ^1^H/ppm	p_value	|log_2_FC|	Type
1	C_4_H_7_O_2_^-^	Butyrate	Bu	0.90(t),1.56(m),2.15(t)	0.00125	2.1934	UP
2	C_6_H_13_NO_2_	Isoleucine	lle	1.01(d),0.99(d)	0.002175	2.5217	UP
3	C_5_H_11_NO_2_	Valine	Val	1.04(d)	0.000428	2.8211	UP
4	C_3_H_5_O_2_^-^	Propionate	Prop	1.06(t)	0.001121	2.4208	UP
5	C_3_H_5_O_3_^-^	Lactate	Lac	1.33(d)	0.017565	1.3096	UP
6	C_6_H_14_N_2_O_2_	Lysine	Lys	1.73(m),3.76(t)	0.009568	2.0104	UP
7	C_6_H_13_NO_2_	Leucine	Leu	1.72(m)	0.007206	2.0666	UP
8	C_2_H_3_O_2_^-^	Acetate	Ace	1.92(s)	0.000206	2.3516	UP
9	C_5_H_11_NO_2_S	Methionine	Met	2.14(s)	0.00331	1.6129	UP
10	C_2_H_7_NO_3_S	Taurine	Tau	3.43(t)	0.00027	2.7857	UP
11	C_2_H_5_NO_2_	Glycine	Gly	3.57(s)	0.002631	2.516	UP
12	C_4_H_9_NO_3_	Threonine	Thr	3.58(d),1.31(d)	0.002215	2.6053	UP
13	C_6_H_13_N_3_O_3_	Citrulline	Cir	3.75(t)	0.00295	1.0506	UP
14	C_3_H_7_NO_2_	Alanine	Ala	3.78(q),1.49(d)	0.00692	1.161	UP

**Table 4:** The metabolites identified in feces following metformin treatment and their fold change values compared to the PCOS group.

NO.	Formula	Compounds	Abbreviate	δ^1^H/ppm	p_value	|log_2_FC|	Type
1	C_4_H_7_O_2_^-^	Butyrate	Bu	0.90(t)	0.0034632	1.1148	UP
2	C_5_H_11_NO_2_	Valine	Val	1.04(d)	0.00099133	2.0679	UP
3	C_3_H_5_O_2_^-^	Propionate	Prop	1.06(t)	0.0046933	1.0202	UP
4	C_3_H_5_O_3_^-^	Lactate	Lac	1.33(d)	0.015755	0.93906	UP
5	C_6_H_13_NO_2_	Isoleucine	Iso	1.48(m)	0.0046054	0.90883	UP
6	C_6_H_13_NO_2_	Leucine	Leu	1.72(m)	0.0055266	1.2315	UP
7	C_6_H_14_N_2_O_2_	Lysine	Lys	1.73(m)	0.0074863	1.1859	UP
8	C_2_H_3_O_2_^-^	Acetate	Ace	1.92(s)	0.00046555	1.8232	UP
9	C_2_H_5_NO_2_	Glycine	Gly	3.57(s)	0.0067228	1.6334	UP
10	C_4_H_9_NO_3_	Threonine	Thr	3.58(d)	0.00090854	2.1984	UP

**Figure 7:**
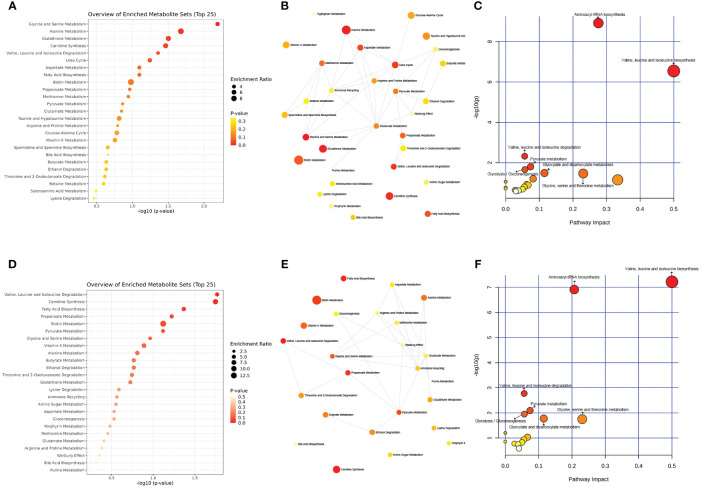
The metabolic pathways associated with moxibustion treatment in PCOS rats. **(A)** Enriched analysis of moxibustion treatment in PCOS rats. **(B, C)** Pathway analysis of moxibustion treatment in PCOS rats. **(D)** Enriched analysis of metformin treatment in PCOS rats. **(E, F)** Pathway analysis of metformin treatment in PCOS rats. Data are mean ± s.d. n = 6.

Further analysis using the MetaboAnalyst website (https://www.metaboanalyst.ca/) and the existing human metabolome database (https://hmdb.ca/) explored the endogenous differential metabolites produced in the PCOS-induced rat faeces under moxibustion and metformin treatment. The metabolic pathways associated with the differential metabolites produced by moxibustion and metformin treatment were found to be similar, including Aminoacyl-tRNA biosynthesis; Valine, leucine, and isoleucine biosynthesis; Valine, leucine, and isoleucine degradation; Pyruvate metabolism; Glycolysis/Gluconeogenesis; Glyoxylate and dicarboxylate metabolism; Glycine, serine, and threonine metabolism (**Figures 7B, C, E, F**). Enrichment analysis revealed that moxibustion affected the Glycine and Serine Metabolism; Alanine Metabolism; Glutathione Metabolism; Carnitine Synthesis; Valine, Leucine, and Isoleucine Degradation pathways in faeces (**Figures 7A, D**). Overall, the metabolic mechanisms of moxibustion therapy for PCOS might be similar to metformin treatment, involving carbohydrate metabolism, amino acid metabolism, and translation.

### 3.4 Associations between gut microbiota and metabolites

**Figure 8:**
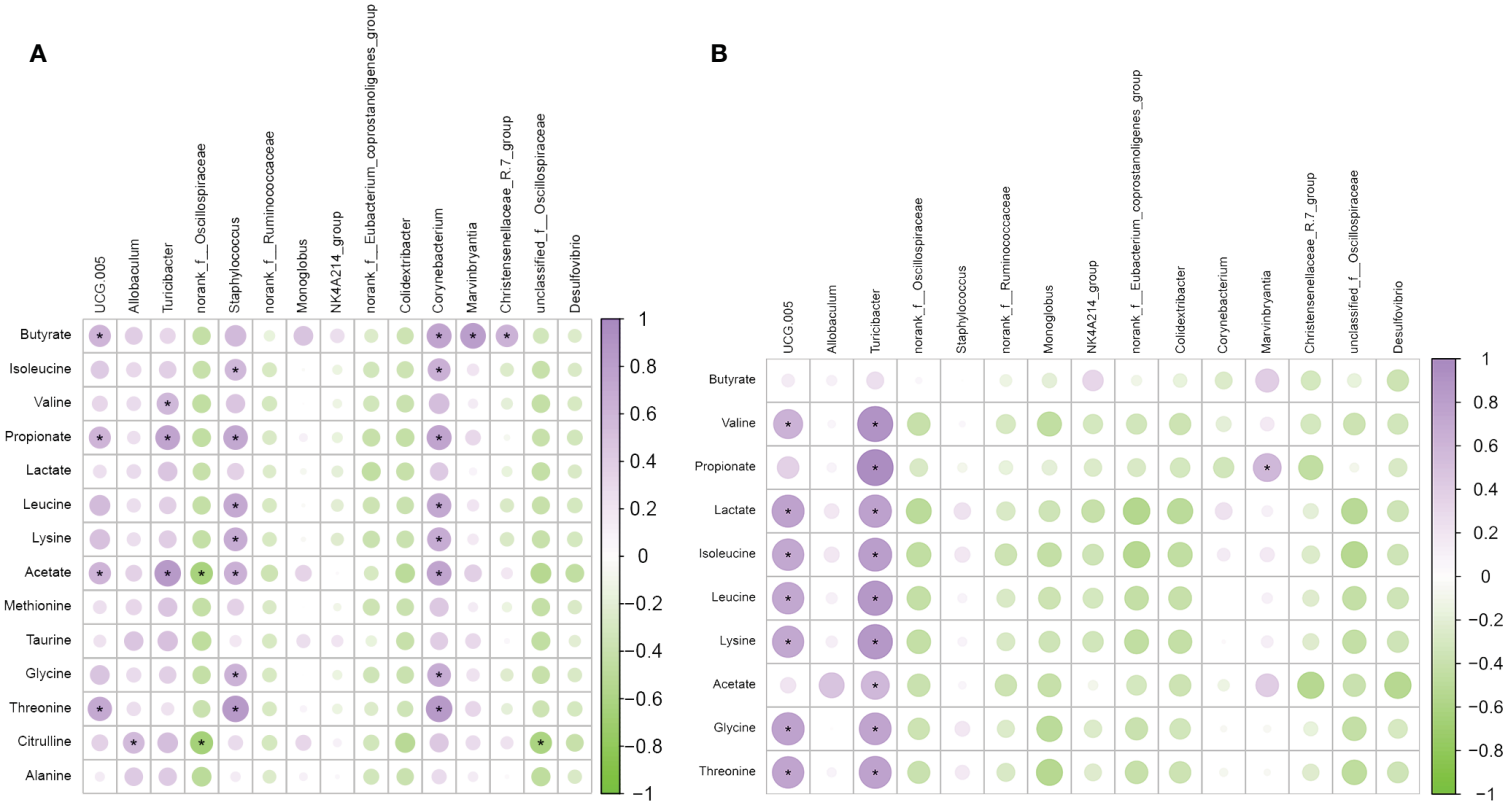
Associations between gut microbiota and metabolites. **(A)** The correlation analysis of metabolites and microbiota in the PCOS Group and MBT Group. **(B)** The correlation analysis of metabolites and microbiota in the PCOS Group and MET Group. Data are mean ± s.d. n = 6. * *p* < 0.05.

For in-depth research of connection between the abundance of circulating metabolites and the gut microbiota influenced by moxibustion, we conducted Spearman analysis to investigate the correlation between 15 genera and these metabolites (**Figures 8A, B**). Among the metabolites resulting from moxibustion treatment, namely lactate, alanine, and methionine, there were no statistically significant correlations observed with any of the genera. Conversely, in the case of metabolites produced by metformin treatment, specifically butyrate, there was no significant correlation found with any of the genera. Subsequently, our investigation turned towards the metabolites influenced by moxibustion. Among these metabolites, which encompass isoleucine, valine, taurine, and glycine, we observed notable correlations with one or two genera. In contrast, a set of seven metabolites, specifically butyrate, propionate, leucine, lysine, acetate, threonine, and citrulline, displayed substantial correlations with a minimum of three genera. In the final phase of our analysis, we scrutinized the metabolites affected by metformin treatment. Within this category, we considered five metabolites. In this context, two or three genera displayed significant correlations, with two metabolites revealing significant correlations with as many as four genera.

In summary, Spearman analysis unveiled varying degrees of correlation between the abundance of circulating metabolites and specific genera influenced by moxibustion treatment, shedding light on the complex interactions within the gut microbiota-metabolite network.

## 4 Discussion

PCOS patients typically exhibit elevated androgen levels (Zeng et al., 2020). Elevated androgen levels are linked to the pathophysiology of PCOS, promoting the simultaneous development of multiple follicles in the ovaries. These follicles often fail to ovulate normally, leading to the formation of ovarian cysts and the onset of PCOS. Moreover, elevated androgen levels can lead to insulin resistance, increasing the risk of metabolic disorders in PCOS patients (Cadagan et al., 2016; Zhang et al., 2016). IR can also trigger further excess androgen production, creating a vicious cycle. Additionally, research (Zhang et al., 2019a) suggests that androgens, in inducing phenotypes resembling PCOS, can disrupt the gut microbiota balance in rodents. These findings have been validated in rat models of PCOS, where DHEA, an androgen originating from the adrenal glands (Poojary et al., 2022), was administered to mimic the development of PCOS. In this study, we have demonstrated the multifaceted effects of moxibustion in a DHEA-induced PCOS rat model, effectively reducing body weight, promoting follicle development and maturation, enhancing insulin sensitivity, regulating gut microbiota, and ameliorating metabolic disorders, thus improving ovarian dysfunction in PCOS rats.

The combined effects of insulin resistance and elevated androgen levels are likely the primary contributors to PCOS (Ding et al., 2021). Research indicates that (Rosenfield and Ehrmann, 2016) insulin plays a role in driving excess androgen production, serving as a gonadotropin for androgens. Excess insulin may lead to elevated androgen levels (Baillargeon and Carpentier, 2007), a phenomenon validated in animal experiments. Insulin signaling can directly impact androgen production (Wu et al., 2014) or stimulate the release of gonadotropins from the pituitary and hypothalamus (Adashi et al., 1981), inducing abnormal androgen production and ovarian dysfunction. Moreover, elevated androgen levels can disrupt metabolism, potentially affecting ovarian development and increasing insulin resistance, ultimately giving rise to the metabolic features of PCOS (Abi Salloum et al., 2015). This endocrine and metabolic dysregulation not only leads to PCOS but also plays a crucial role in causing infertility by disrupting the normal ovulation process and egg development, thus affecting women’s fertility. Insulin resistance and hyperandrogenism can create a vicious cycle, exacerbating PCOS symptoms and increasing infertility risks. Moxibustion is a traditional treatment for gynaecological disorders, which has shown efficacy in improving symptoms of PCOS in clinical trials. Furthermore, researchers have found that moxibustion can enhance the success rate of *in vitro* fertilization-embryo transfer (IVF-ET) treatment by improving endometrial blood flow, morphology, and hormone levels (Chen and Hau, 2015). Our experimental results demonstrate that, in comparison to the PCOS group, moxibustion intervention significantly improved androgen levels, insulin resistance, and ovarian tissue pathology. This suggests that moxibustion can ameliorate androgen levels and regulate insulin resistance as a treatment for PCOS. Metformin, a type of insulin sensitizer, is commonly used as a first-line anti-insulin resistance medication (Sanchez-Rangel and Inzucchi, 2017). Research suggests that metformin can effectively enhance insulin sensitivity and appears to mitigate insulin-mediated androgen production, alleviating hyperandrogenism in PCOS patients and improving ovarian function (Velazquez et al., 1994; Palomba et al., 2009; Pauli et al., 2011). Consequently, in this study, metformin was selected as the positive control agent for the treatment of PCOS. In the current study, there were no significant differences in biochemical indicators such as FINS, FBG, HOMA-IR, and ISI between the MET and MBT groups.

In recent years, numerous studies have emphasized a close association between microbial dysbiosis and PCOS (Torres et al., 2018; Jobira et al., 2020; Yang et al., 2021). Some researchers (Deng, 2019) conducted experiments involving the transplantation of gut microbiota from PCOS patients into mice. The experimental findings suggested that dysbiosis of the gut microbiome could trigger the development of PCOS, potentially serving as one of the contributing factors in PCOS pathogenesis. Additionally, research indicates that disruptions in the gut microbiota would lead to the production of lipopolysaccharides (LPS) and alterations in intestinal mucosal permeability (Tremellen and Pearce, 2012). LPS, known for its endotoxin properties, can interact with toll-like receptors on the surface of intestinal epithelial cells, thereby activating the nuclear factor κB (NF-κB) pathway and triggering an inflammatory response (Yurtdaş and Akdevelioğlu, 2020). Some researchers have also found that the gut microbiota can mediate insulin resistance through LPS (Ganie et al., 2019). This process is closely associated with the chronic inflammatory state observed in PCOS patients (Zhu et al., 2016), highlighting the substantial connection between gut microbiota and metabolic health. In this study, an analysis of gut microbiota diversity indicated a tendency towards increased α-diversity in DHEA-induced PCOS rats. While this aligns with previous research findings, it did not reach statistical significance (Zhu et al., 2020; Yang et al., 2021). Furthermore, moxibustion has obvious impact on the gut microbiota, resulting in substantial changes in the microbial communities associated with PCOS. We observed marked disparities in the gut microbiota composition between the CTRL group and the DHEA-intervened rats, with moxibustion treatment partially mitigating some of these distinctions. Notably, *Desulfovibrio* (Moreno-Indias et al., 2016) a bacterium known for generating Gram-negative endotoxins associated with heightened gut permeability and gut-derived antigens, had previously been observed in elevated levels in the intestines of DHEA-induced PCOS mice (Huang et al., 2022). In our study, we similarly detected increased levels of *Desulfovibrio* in the PCOS group, which significantly decreased following moxibustion treatment. These findings imply that *Desulfovibrio* may be one of the principal pathogens contributing to PCOS, and moxibustion may impede the proliferation of harmful bacteria. In the MBT group, the prevalence of the *UCG-005* genus was significantly higher than in the PCOS group. *UCG-005* is believed to play a pivotal role in preserving gastrointestinal mucosal barriers and preventing metabolic disorders associated with IR (Chen et al., 2021). It has the capacity to enhance mitochondrial activity, improve energy metabolism, and stimulate intestinal gluconeogenesis, thereby inducing beneficial metabolic effects (Hartstra et al., 2015). Currently, *UCG-005* is regarded as a probiotic that holds promise for the prevention and treatment of obesity, type 2 diabetes, and other metabolic disorders. Furthermore, clinical research (Chen et al., 2021) has substantiated a negative correlation between the abundance of *UCG-005* and IR, as well as the risk of type 2 diabetes in patients. Hence, the potential impact of moxibustion on PCOS through the gut microbiota might be associated with the increased prevalence of *UCG-005*. Consequently, moxibustion could potentially ameliorate PCOS in rats, at least partially, by improving the gut microbiota. It is imperative to note that the bacterial composition in faeces may not comprehensively reflect the overall alterations in the host’s gut microbiota. *Turicibacter* is a crucial constituent of the gut microbiota closely intertwined with host metabolic shifts (Browne et al., 2016; Jiao et al., 2018). Research has indicated that *Turicibacter* modifies host bile acids and lipid metabolism (Lynch et al., 2023). In our investigation, *Turicibacter* exhibited significantly higher levels in the MET group compared to the PCOS group, suggesting that moxibustion might influence PCOS through interactions with specific metabolites via the gut microbiota. Further exploration using metabolomics approaches is warranted to thoroughly investigate these interactions.

Metabolomics involves the qualitative and quantitative analysis of blood, urine, faeces, and tissues to enhance our comprehension of specific metabolites, diseases, and their developmental variations (Yang et al., 2005). The human body is increasingly recognized as a superorganism (Sleator, 2010), housing trillions of symbiotic microorganisms both within and around it. These microorganisms interact through specific pathways to produce metabolites, exerting profound effects on the host’s homeostasis (Wang et al., 2018; Schoeler and Caesar, 2019; Zhao et al., 2022). Therefore, we utilize a combination of fecal metabolomics and 16S rRNA gene sequencing to further elucidate the intricate relationship between the microbial community and the host. After conducting ^1^H NMR metabolomics analysis of rat faeces, it is intriguing to note that we found differential metabolites produced in the MET group, which served as a positive control, to be highly similar to those in the MBT group. Furthermore, pathway and enrichment analyses of the differential metabolites in both groups revealed certain similarities in the associated metabolic pathways. This suggests that the metabolic mechanisms of moxibustion therapy for PCOS may bear some resemblance to the treatment with metformin. Simultaneously, microbiome functional prediction analysis (**Figure 4G**) indicates that the gut microbiota may play a pivotal role in influencing metabolic functions, especially in Amino acid transport and metabolism; Translation, ribosomal structure and biogenesis; Transcription, as well as Carbohydrate transport and metabolism, interestingly, this aligns with the pathways affected by differential metabolites.

Through correlation analysis, it was found that the levels of fecal acetic acid, propionic acid, and butyric acid were significantly correlated with the abundance of several genera in the gut microbiota, suggesting that short-chain fatty acids (SCFAs) may be the primary host metabolites interacting with the gut microbiota. Acetic acid, propionic acid, and butyric acid are prominent SCFAs synthesized by intestinal bacteria from various substrates, and they are metabolites derived from the microbial community (Cook and Sellin, 1998). Research indicates that SCFAs are primarily produced by the microbial process of sugar fermentation (Macfarlane and Macfarlane, 2003), which aligns with the metabolic pathways affected by moxibustion or metformin treatment for PCOS. Additionally, small amounts of SCFAs can also arise during the catabolism of branched-chain amino acids like valine, leucine, and isoleucine. It’s well-known that SCFAs have various impacts on host physiological functions, including improving energy metabolism, particularly by regulating disruptions in glucose and lipid metabolism (He et al., 2020). Previous studies have suggested that SCFAs, especially butyric acid (Gao et al., 2009), stimulate the release of glucagon-like peptide-1 (GLP-1) in mice through the activation of the G protein-coupled receptor FFAR2 (Tolhurst et al., 2012), and GLP-1 promotes insulin secretion and enhances insulin sensitivity in the body (Meier, 2012). Additionally, research on db/db mice found that a complex probiotic supplement could can augment GLP-1 secretion by increasing levels of SCFA-producing bacteria and SCFAs themselves (Wang et al., 2020). Furthermore, SCFAs can influence the secretion of regulatory hormones. Experiments have shown that SCFAs affect the synthesis and secretion of progesterone and estradiol in porcine ovarian granulosa cells through the cAMP-PKA pathway mediated by GPR41 and GPR43 (Li, 2015). In a high androgen environment, SCFAs can protect the body from oxidative stress-induced tissue damage by blocking androgen receptors and mineralocorticoid receptors (Usman et al., 2021). Clinical investigations have also indicated that the interaction between the gut microbiota and SCFAs may play a crucial role in the regulation of sex hormones in PCOS patients (Zhang et al., 2019b). In our study, we observed a substantial increase in the concentrations of acetic acid, propionic acid, and butyric acid after moxibustion treatment in rats with DHEA-induced PCOS. Furthermore, moxibustion treatment led to a positive correlation between the differential fecal microbiota *UCG-005* and *Turicibacter* and fecal metabolites SCFAs (acetic acid, propionic acid, and butyric acid), while *Desulfovibrio* showed a negative correlation. Interestingly, *UCG-005* is a producer of SCFAs, whereas *Desulfovibrio* is known for producing LPS. Based on these observations, we hypothesize that the gut microbiota of rats with DHEA-induced PCOS undergoes significant changes following moxibustion treatment, promoting the production of metabolites such as SCFAs. Through the combined action of the gut microbiota and metabolites, insulin resistance and metabolic disturbances in rats with PCOS are improved.

However, it’s important to note that SCFAs primarily come from plant-based foods (Dalile et al., 2019), and moxibustion, as an external thermal stress therapy, may promote the production of SCFAs under heat stress conditions (Wang et al., 2016). Nevertheless, further research is imperative to elucidate the precise mechanisms by which moxibustion affects the gut microbiota-metabolism in PCOS rats.

## 5 Conclusion

Our research indicates that moxibustion can facilitate the restoration of ovarian dysfunction, improve insulin resistance-related markers, influence the abundance of *UCG-005*, *Turicibacter*, and *Desulfovibrio*, and promote elevated levels of short-chain fatty acids (acetic acid, propionic acid, and butyric acid) associated with gut microbiota.

## Data availability statement

The datasets presented in this study can be found in online repositories. The names of the repository/repositories and accession number(s) can be found below: https://www.ncbi.nlm.nih.gov/sra/PRJNA1032518.

## Ethics statement

The animal study was approved by The Xiamen University Experimental Animal Center Ethics Committee. The study was conducted in accordance with the local legislation and institutional requirements.

## Author contributions

YL: Writing – original draft, Writing – review & editing, Data curation, Methodology, Project administration, Software, Validation, Visualization. HZ: Writing – original draft, Writing – review & editing, Methodology, Supervision, Validation, Visualization. JL: Writing – review & editing, Supervision, Visualization. YP: Supervision, Writing – review & editing. XQ: Supervision, Writing – review & editing. LW: Writing – review & editing, Data curation, Supervision, Visualization. LC: Supervision, Writing – review & editing. NB: Writing – review & editing, Data curation, Funding acquisition, Investigation, Supervision.

## References

[B1] Abi Salloum B. Veiga-Lopez A. Abbott D. H. Burant C. F. Padmanabhan V. (2015). Developmental programming: exposure to testosterone excess disrupts steroidal and metabolic environment in pregnant sheep. Endocrinology 156, 2323–2337. doi: 10.1210/en.2014-2006 25763641 PMC4430607

[B2] Adashi E. Y. Hsueh A. J. Yen S. S. (1981). Insulin enhancement of luteinizing hormone and follicle-stimulating hormone release by cultured pituitary cells. Endocrinology 108, 1441–1449. doi: 10.1210/endo-108-4-1441 6781875

[B3] Amisi C. A. (2022). Markers of insulin resistance in Polycystic ovary syndrome women: An update. World J. Diabetes 13, 129–149. doi: 10.4239/wjd.v13.i3.129 35432749 PMC8984569

[B4] Baillargeon J. P. Carpentier A. (2007). Role of insulin in the hyperandrogenemia of lean women with polycystic ovary syndrome and normal insulin sensitivity. Fertil Steril 88, 886–893. doi: 10.1016/j.fertnstert.2006.12.055 17559844 PMC3846535

[B5] Bayona A. Martínez-Vaello V. Zamora J. Nattero-Chávez L. Luque-Ramírez M. Escobar-Morreale H. F. (2022). Prevalence of PCOS and related hyperandrogenic traits in premenopausal women with type 1 diabetes: a systematic review and meta-analysis. Hum. Reprod. Update 28, 501–517. doi: 10.1093/humupd/dmac011 35237802

[B6] Browne H. P. Forster S. C. Anonye B. O. Kumar N. Neville B. A. Stares M. D.. (2016). Culturing of 'unculturable' human microbiota reveals novel taxa and extensive sporulation. Nature 533, 543–546. doi: 10.1038/nature17645 27144353 PMC4890681

[B7] Cadagan D. Khan R. Amer S. (2016). Thecal cell sensitivity to luteinizing hormone and insulin in polycystic ovary syndrome. Reprod. Biol. 16, 53–60. doi: 10.1016/j.repbio.2015.12.006 26952754

[B8] Chen Q. Hau C. (2015). [Impacts on pregnancy outcome treated with acupuncture and moxibustion in IVF-ET patients]. Zhongguo Zhen Jiu 35, 313–317. doi: 10.13703/j.0255-2930.2015.04.001 26054135

[B9] Chen Y. Yang T. Hao C. Zhao J. (2018). A retrospective study of letrozole treatment prior to human chorionic gonadotropin in women with polycystic ovary syndrome undergoing *in vitro* fertilization at risk of ovarian hyperstimulation syndrome. Med. Sci. Monit 24, 4248–4253. doi: 10.12659/MSM.910743 29925074 PMC6042308

[B10] Chen Z. Radjabzadeh D. Chen L. Kurilshikov A. Kavousi M. Ahmadizar F.. (2021). Association of insulin resistance and type 2 diabetes with gut microbial diversity: A microbiome-wide analysis from population studies. JAMA Netw. Open 4, e2118811. doi: 10.1001/jamanetworkopen.2021.18811 34323983 PMC8322996

[B11] Cook S. I. Sellin J. H. (1998). Review article: short chain fatty acids in health and disease. Aliment Pharmacol. Ther. 12, 499–507. doi: 10.1046/j.1365-2036.1998.00337.x 9678808

[B12] Dalile B. Van Oudenhove L. Vervliet B. Verbeke K. (2019). The role of short-chain fatty acids in microbiota-gut-brain communication. Nat. Rev. Gastroenterol. Hepatol. 16, 461–478. doi: 10.1038/s41575-019-0157-3 31123355

[B13] Deng Y. (2019). Preliminary Exploration of the Role of Gut Microbiota Dysbiosis in the Pathogenesis of Polycystic Ovary Syndrome. (China: Peking Union Medical College). dissertation.

[B14] Ding H. Zhang J. Zhang F. Zhang S. Chen X. Liang W.. (2021). Resistance to the insulin and elevated level of androgen: A major cause of polycystic ovary syndrome. Front. Endocrinol. (Lausanne) 12. doi: 10.3389/fendo.2021.741764 PMC8564180 34745009

[B15] Gan J. Chen J. Ma R. L. Deng Y. Ding X. S. Zhu S. Y.. (2023). Metagenomics study on taxonomic and functional change of gut microbiota in patients with obesity with PCOS treated with exenatide combination with metformin or metformin alone. Gynecol Endocrinol. 39, 2219342. doi: 10.1080/09513590.2023.2219342 37290480

[B16] Ganie M. A. Sahar T. Rashid A. Wani I. A. Nisar S. Sathyapalan T.. (2019). Comparative evaluation of biomarkers of inflammation among Indian women with polycystic ovary syndrome (PCOS) consuming vegetarian vs. Non-vegetarian diet. Front. Endocrinol. (Lausanne) 10. doi: 10.3389/fendo.2019.00699 PMC6857098 31781027

[B17] Gao Z. Yin J. Zhang J. Ward R. E. Martin R. J. Lefevre M.. (2009). Butyrate improves insulin sensitivity and increases energy expenditure in mice. Diabetes 58, 1509–1517. doi: 10.2337/db08-1637 19366864 PMC2699871

[B18] Guo X. Okpara E. S. Hu W. Yan C. Wang Y. Liang Q.. (2022). Interactive relationships between intestinal flora and bile acids. Int. J. Mol. Sci. 23 (15), 8343. doi: 10.3390/ijms23158343 35955473 PMC9368770

[B19] Hamilton K. P. Zelig R. Parker A. R. Haggag A. (2019). Insulin resistance and serum magnesium concentrations among women with polycystic ovary syndrome. Curr. Dev. Nutr. 3, nzz108. doi: 10.1093/cdn/nzz108 31696157 PMC6822014

[B20] Hartstra A. V. Bouter K. E. Bäckhed F. Nieuwdorp M. (2015). Insights into the role of the microbiome in obesity and type 2 diabetes. Diabetes Care 38, 159–165. doi: 10.2337/dc14-0769 25538312

[B21] He J. Zhang P. Shen L. Niu L. Tan Y. Chen L.. (2020). Short-chain fatty acids and their association with signalling pathways in inflammation, glucose and lipid metabolism. Int. J. Mol. Sci. 21 (17), 6356. doi: 10.3390/ijms21176356 32887215 PMC7503625

[B22] Hu M. H. Zheng S. X. Yin H. Zhu X. Y. Lu F. T. Tong X. H.. (2020). Identification of microRNAs that regulate the MAPK pathway in human cumulus cells from PCOS women with insulin resistance. Reprod. Sci. 27, 833–844. doi: 10.1007/s43032-019-00086-5 32046427

[B23] Huang J. Chen P. Xiang Y. Liang Q. Wu T. Liu J.. (2022). Gut microbiota dysbiosis-derived macrophage pyroptosis causes polycystic ovary syndrome via steroidogenesis disturbance and apoptosis of granulosa cells. Int. Immunopharmacol 107, 108717. doi: 10.1016/j.intimp.2022.108717 35334358

[B24] Jiao N. Baker S. S. Nugent C. A. Tsompana M. Cai L. Wang Y.. (2018). Gut microbiome may contribute to insulin resistance and systemic inflammation in obese rodents: a meta-analysis. Physiol. Genomics 50, 244–254. doi: 10.1152/physiolgenomics.00114.2017 29373083

[B25] Jobira B. Frank D. N. Pyle L. Silveira L. J. Kelsey M. M. Garcia-Reyes Y.. (2020). Obese adolescents with PCOS have altered biodiversity and relative abundance in gastrointestinal microbiota. J. Clin. Endocrinol. Metab. 105, e2134–e2144. doi: 10.1210/clinem/dgz263 31970418 PMC7147870

[B26] Li M. J. (2015). Short Chain Fatty Acids Regulating Steroid Hormones Secretion Through cAMP-PKA Signaling on Porcine Granulosa Cell. (China: Nanjing Agricultural University). dissertation.

[B27] Liao B. Qiao J. Pang Y. (2021). Central regulation of PCOS: abnormal neuronal-reproductive-metabolic circuits in PCOS pathophysiology. Front. Endocrinol. (Lausanne) 12. doi: 10.3389/fendo.2021.667422 PMC8194358 34122341

[B28] Lin X. W. (2022). Comparative Study on Biosample Preparation and Data Pretreatment for NMR-based Metabolomics. (China: Xiamen University). master's thesis.

[B29] Liu Z. J. (2013). Chinese Veterinary Acupuncture and Moxibustion (China: China Agriculture Press).

[B30] Liu K. He X. Huang J. Yu S. Cui M. Gao M.. (2023a). Short-chain fatty acid-butyric acid ameliorates granulosa cells inflammation through regulating METTL3-mediated N6-methyladenosine modification of FOSL2 in polycystic ovary syndrome. Clin. Epigenet. 15, 86. doi: 10.1186/s13148-023-01487-9 PMC10183145 37179374

[B31] Liu Y. Xu R. Zhou Y. Wang Y. Zhang F. Tong X.. (2023b). Diane-35 and metformin therapy in rats with endometrial lesions induced by dihydrotestosterone exposure. Ann. Transl. Med. 11, 247. doi: 10.21037/atm-21-2441 37082665 PMC10113095

[B32] Liu C. Zhao D. Ma W. Guo Y. Wang A. Wang Q.. (2016). Denitrifying sulfide removal process on high-salinity wastewaters in the presence of Halomonas sp. Appl. Microbiol. Biotechnol. 100, 1421–1426. doi: 10.1007/s00253-015-7039-6 26454867

[B33] Lynch J. B. Gonzalez E. L. Choy K. Faull K. F. Jewell T. Arellano A.. (2023). Gut microbiota Turicibacter strains differentially modify bile acids and host lipids. Nat. Commun. 14, 3669. doi: 10.1038/s41467-023-39403-7 37339963 PMC10281990

[B34] Macfarlane S. Macfarlane G. T. (2003). Regulation of short-chain fatty acid production. Proc. Nutr. Soc. 62, 67–72. doi: 10.1079/PNS2002207 12740060

[B35] Meier J. J. (2012). GLP-1 receptor agonists for individualized treatment of type 2 diabetes mellitus. Nat. Rev. Endocrinol. 8, 728–742. doi: 10.1038/nrendo.2012.140 22945360

[B36] Moreno-Indias I. Torres M. Sanchez-Alcoholado L. Cardona F. Almendros I. Gozal D.. (2016). Normoxic recovery mimicking treatment of sleep apnea does not reverse intermittent hypoxia-induced bacterial dysbiosis and low-grade endotoxemia in mice. Sleep 39, 1891–1897. doi: 10.5665/sleep.6176 27397563 PMC5020371

[B37] Paixão L. Ramos R. B. Lavarda A. Morsh D. M. Spritzer P. M. (2017). Animal models of hyperandrogenism and ovarian morphology changes as features of polycystic ovary syndrome: a systematic review. Reprod. Biol. Endocrinol. 15, 12. doi: 10.1186/s12958-017-0231-z 28183310 PMC5301391

[B38] Palomba S. (2021). Is fertility reduced in ovulatory women with polycystic ovary syndrome? An opinion paper. Hum. Reprod. 36, 2421–2428. doi: 10.1093/humrep/deab181 34333641

[B39] Palomba S. Daolio J. La Sala G. B. (2017). Oocyte competence in women with polycystic ovary syndrome. Trends Endocrinol. Metab. 28, 186–198. doi: 10.1016/j.tem.2016.11.008 27988256

[B40] Palomba S. de Wilde M. A. Falbo A. Koster M. P. La Sala G. B. Fauser B. C. (2015). Pregnancy complications in women with polycystic ovary syndrome. Hum. Reprod. Update 21, 575–592. doi: 10.1093/humupd/dmv029 26117684

[B41] Palomba S. Falbo A. Zullo F. Orio F. Jr. (2009). Evidence-based and potential benefits of metformin in the polycystic ovary syndrome: a comprehensive review. Endocr. Rev. 30, 1–50. doi: 10.1210/er.2008-0030 19056992

[B42] Palomba S. Piltonen T. T. Giudice L. C. (2021). Endometrial function in women with polycystic ovary syndrome: a comprehensive review. Hum. Reprod. Update 27, 584–618. doi: 10.1093/humupd/dmaa051 33302299

[B43] Pauli J. M. Raja-Khan N. Wu X. Legro R. S. (2011). Current perspectives of insulin resistance and polycystic ovary syndrome. Diabetes Med. 28, 1445–1454. doi: 10.1111/j.1464-5491.2011.03460.x 21950959

[B44] Poojary P. S. Nayak G. Panchanan G. Rao A. Kundapur S. D. Kalthur S. G.. (2022). Distinctions in PCOS induced by letrozole vs dehydroepiandrosterone with high-fat diet in mouse model. Endocrinology 163 (9), bqac097. doi: 10.1210/endocr/bqac097 35776497

[B45] Ramu S. K. Praveen Ankith Yadav K. (2022). Study of diversity of metformin related gastrointestinal side effects. J. Assoc. Physicians India 70, 11–12. 35833410

[B46] Reutov V. P. Sorokina E. G. (2022). Causal relationship between physiological and pathological processes in the brain and in the gastrointestinal tract: the brain-intestine axis. Biophysics (Oxf) 67, 972–986. doi: 10.1134/S0006350922060197 36883179 PMC9984134

[B47] Rosenfield R. L. Ehrmann D. A. (2016). The pathogenesis of polycystic ovary syndrome (PCOS): the hypothesis of PCOS as functional ovarian hyperandrogenism revisited. Endocr. Rev. 37, 467–520. doi: 10.1210/er.2015-1104 27459230 PMC5045492

[B48] Roy S. Mahesh V. B. Greenblatt R. B. (1962). Effect of dehydroepiandrosterone and delta4-androstenedione on the reproductive organs of female rats: production of cystic changes in the ovary. Nature 196, 42–43. doi: 10.1038/196042a0 13982862

[B49] Sanchez-Rangel E. Inzucchi S. E. (2017). Metformin: clinical use in type 2 diabetes. Diabetologia 60, 1586–1593. doi: 10.1007/s00125-017-4336-x 28770321

[B50] Schoeler M. Caesar R. (2019). Dietary lipids, gut microbiota and lipid metabolism. Rev. Endocr. Metab. Disord. 20, 461–472. doi: 10.1007/s11154-019-09512-0 31707624 PMC6938793

[B51] Segata N. Izard J. Waldron L. Gevers D. Miropolsky L. Garrett W. S.. (2011). Metagenomic biomarker discovery and explanation. Genome Biol. 12, R60. doi: 10.1186/gb-2011-12-6-r60 21702898 PMC3218848

[B52] Sleator R. D. (2010). The human superorganism - of microbes and men. Med. Hypotheses 74, 214–215. doi: 10.1016/j.mehy.2009.08.047 19836146

[B53] Tolhurst G. Heffron H. Lam Y. S. Parker H. E. Habib A. M. Diakogiannaki E.. (2012). Short-chain fatty acids stimulate glucagon-like peptide-1 secretion via the G-protein-coupled receptor FFAR2. Diabetes 61, 364–371. doi: 10.2337/db11-1019 22190648 PMC3266401

[B54] Torres P. J. Siakowska M. Banaszewska B. Pawelczyk L. Duleba A. J. Kelley S. T.. (2018). Gut microbial diversity in women with polycystic ovary syndrome correlates with hyperandrogenism. J. Clin. Endocrinol. Metab. 103, 1502–1511. doi: 10.1210/jc.2017-02153 29370410 PMC6276580

[B55] Tremellen K. Pearce K. (2012). Dysbiosis of Gut Microbiota (DOGMA)–a novel theory for the development of Polycystic Ovary Syndrome. Med. Hypotheses 79, 104–112. doi: 10.1016/j.mehy.2012.04.016 22543078

[B56] Usman T. O. Adeyanju O. A. Areola E. D. Badmus O. O. Oyeyipo I. P. Olaniyi K. S.. (2021). Acetate causes renoprotection like androgen and mineralocorticoid receptors blockade in testosterone-exposed pregnant rats. Mol. Cell Biochem. 476, 1861–1870. doi: 10.1007/s11010-020-04031-y 33479808

[B57] Velazquez E. M. Mendoza S. Hamer T. Sosa F. Glueck C. J. (1994). Metformin therapy in polycystic ovary syndrome reduces hyperinsulinemia, insulin resistance, hyperandrogenemia, and systolic blood pressure, while facilitating normal menses and pregnancy. Metabolism 43, 647–654. doi: 10.1016/0026-0495(94)90209-7 8177055

[B58] Wang Y. Dilidaxi D. Wu Y. Sailike J. Sun X. Nabi X. H. (2020). Composite probiotics alleviate type 2 diabetes by regulating intestinal microbiota and inducing GLP-1 secretion in db/db mice. BioMed. Pharmacother. 125, 109914. doi: 10.1016/j.biopha.2020.109914 32035395

[B59] Wang Z. Huang H. Xu Q. Li J. (2022). Application of disodium terephthalate as an internal standard in the quantitative NMR-based metabolomics. J. Xiamen Univ. Natural Sci. 61, 7. doi: 10.6043/j.issn.0438-0479.202103034

[B60] Wang L. Urriola P. E. Luo Z. H. Rambo Z. J. Wilson M. E. Torrison J. L.. (2016). Metabolomics revealed diurnal heat stress and zinc supplementation-induced changes in amino acid, lipid, and microbial metabolism. Physiol. Rep. 4 (1), e12676. doi: 10.14814/phy2.12676 26755737 PMC4760408

[B61] Wang J. Wang C. Liu H. Qi H. Chen H. Wen J. (2018). Metabolomics assisted metabolic network modeling and network wide analysis of metabolites in microbiology. Crit. Rev. Biotechnol. 38, 1106–1120. doi: 10.1080/07388551.2018.1462141 29683004

[B62] Wang J. Wu D. Guo H. Li M. (2019). Hyperandrogenemia and insulin resistance: The chief culprit of polycystic ovary syndrome. Life Sci. 236, 116940. doi: 10.1016/j.lfs.2019.116940 31604107

[B63] Wu S. Divall S. Nwaopara A. Radovick S. Wondisford F. Ko C.. (2014). Obesity-induced infertility and hyperandrogenism are corrected by deletion of the insulin receptor in the ovarian theca cell. Diabetes 63, 1270–1282. doi: 10.2337/db13-1514 24379345 PMC3964497

[B64] Xu K. Wang J. Hu F. Lv S. Zhang Y. Yang Q.. (2021). Effects of moxibustion on reproduction and metabolism of polycystic ovary syndrome: a protocol for meta-analysis and systematic review. BMJ Open 11, e049039. doi: 10.1136/bmjopen-2021-049039 PMC8388304 34433602

[B65] Yang J. Song S. L. Castro-Perez J. Plumb R. S. Xu G. W. (2005). [Metabonomics and its applications]. Sheng Wu Gong Cheng Xue Bao 21, 1–5. doi: 10.3321/j.issn:1000-3061.2005.01.001 15859320

[B66] Yang Y. L. Zhou W. W. Wu S. Tang W. L. Wang Z. W. Zhou Z. Y.. (2021). Intestinal flora is a key factor in insulin resistance and contributes to the development of polycystic ovary syndrome. Endocrinology 162 (10), bqab118. doi: 10.1210/endocr/bqab118 34145455 PMC8375444

[B67] Yurtdaş G. Akdevelioğlu Y. (2020). A new approach to polycystic ovary syndrome: the gut microbiota. J. Am. Coll. Nutr. 39, 371–382. doi: 10.1080/07315724.2019.1657515 31513473

[B68] Zeng X. Xie Y. J. Liu Y. T. Long S. L. Mo Z. C. (2020). Polycystic ovary syndrome: Correlation between hyperandrogenism, insulin resistance and obesity. Clin. Chim. Acta 502, 214–221. doi: 10.1016/j.cca.2019.11.003 31733195

[B69] Zhang F. Ma T. Cui P. Tamadon A. He S. Huo C.. (2019a). Diversity of the gut microbiota in dihydrotestosterone-induced PCOS rats and the pharmacologic effects of diane-35, probiotics, and berberine. Front. Microbiol. 10. doi: 10.3389/fmicb.2019.00175 PMC6375883 30800111

[B70] Zhang J. Sun Z. Jiang S. Bai X. Ma C. Peng Q.. (2019b). Probiotic Bifidobacterium lactis V9 Regulates the Secretion of Sex Hormones in Polycystic Ovary Syndrome Patients through the Gut-Brain Axis. mSystems 4 (2), e00017-19. doi: 10.1128/mSystems.00017-19 31020040 PMC6469956

[B71] Zhang Y. Sun X. Sun X. Meng F. Hu M. Li X.. (2016). Molecular characterization of insulin resistance and glycolytic metabolism in the rat uterus. Sci. Rep. 6, 30679. doi: 10.1038/srep30679 27461373 PMC4962087

[B72] Zhao L. Wang C. Peng S. Zhu X. Zhang Z. Zhao Y.. (2022). Pivotal interplays between fecal metabolome and gut microbiome reveal functional signatures in cerebral ischemic stroke. J. Transl. Med. 20, 459. doi: 10.1186/s12967-022-03669-0 36209079 PMC9548195

[B73] Zhu Y. Li Y. Liu M. Hu X. Zhu H. (2020). Guizhi fuling wan, chinese herbal medicine, ameliorates insulin sensitivity in PCOS model rats with insulin resistance via remodeling intestinal homeostasis. Front. Endocrinol. (Lausanne) 11. doi: 10.3389/fendo.2020.00575 PMC7482315 32973686

[B74] Zhu Q. Zhou H. Zhang A. Gao R. Yang S. Zhao C.. (2016). Serum LBP is associated with insulin resistance in women with PCOS. PloS One 11, e0145337. doi: 10.1371/journal.pone.0145337 26799617 PMC4723331

